# The role of glomerular lesions in the prognosis of patients with acute kidney injury during hemorrhagic fever with renal syndrome

**DOI:** 10.1080/0886022X.2023.2196349

**Published:** 2023-04-04

**Authors:** Min Min, Meiling Liu, Chunyu Lu, Lina Zhu, Jiong Zhang, Jinquan Wang

**Affiliations:** a National Clinical Research Center of Kidney Diseases, Jingling Hospital, Nanjing Medical University, Nanjing, China; b Department of Nephrology, Anqing First People’s Hospital of Anhui Medical University, Anqing, China

**Keywords:** Hemorrhagic fever with renal syndrome, acute kidney injury, glomerular lesion, Hantavirus, prognosis

## Abstract

**Objective:**

This study aimed to explore the role of glomerular lesions in patients who suffered from acute kidney injury (AKI) during hemorrhagic fever with renal syndrome (HFRS).

**Methods:**

The study comprised 66 patients with AKI during HFRS treated at the National Clinical Research Center of Kidney Diseases of China, Jinling Hospital, from January 2014 to December 2018. According to the kidney pathological findings, the 66 patients were divided into two groups: the tubulointerstitial injury group (HFRS-TI group, *n* = 43) and the tubulointerstitial injury with glomerular lesions group (HFRS-GL group, *n* = 23). The clinical and pathological characteristics of the 66 patients were analyzed.

**Results:**

There were 9 cases of IgA nephropathy, 1 case of membranous nephropathy, 2 cases of diabetic nephropathy, and 11 cases of mesangial proliferative glomerulonephritis in the HFRS-GL group. There were more males in the HFRS-GL group than in the HFRS-TI group (92.3% vs. 69.8%, *p* < .05). A higher proportion of interstitial fibrosis (56.5% vs. 27.9%, *p* < .05) and more immunoglobulin and complement depositions (*p* < .001) were observed in the HFRS-GL group than in the HFRS-TI group. Rates of remission of AKI were lower in the HFRS-GL group than in the HFRS-TI group (73.9% vs. 95.3%, *p* < .05). The presence of glomerular lesions (HR = 5.636, 95% CI = 1.121–28.329, *p* = .036) and moderate tubulointerstitial injury (HR = 3.598, 95% CI = 1.278–10.125, *p* = .015) were found to be independent risk factors for kidney prognosis.

**Conclusions:**

Patients with AKI during HFRS can have glomerular lesions or glomerulonephritis. Patients with AKI during HFRS who have glomerular lesions or moderate renal tubulointerstitial injury proven by kidney biopsy have a relatively poor kidney prognosis. A kidney biopsy can help determine long-term prognosis in patients with AKI during HFRS.

## Introduction

Hemorrhagic fever with renal syndrome (HFRS) is a zoonosis caused by members of the Bunyaviridae virus family, genus Hantavirus, which is carried by rodents and is primarily transmitted through inhaling virus-contaminated aerosols of rodent excrement [1]. It is an acute infectious disease characterized by vascular hemorrhage and can be life-threatening [2]. The major clinical manifestations of HFRS are fever, hemorrhage, hypotension, and acute kidney injury (AKI). Typical patients present with ‘three redness symptoms’ (skin flushing in the face, neck, and upper chest) and ‘three pain symptoms’ (headache, orbital pain, and low back pain). The diagnosis of HFRS is based on typical clinical findings, history of exposure, and serological tests. However, a variety of additional symptoms can occur in most organ systems, and the kidney is the main damaged organ. Although HFRS is a self-limiting disease with gradual recovery from AKI, some patients eventually develop chronic kidney insufficiency [3]. Tubulointerstitial injury is a typical pathological feature of AKI during HFRS. However, we found that some patients with AKI during HFRS had prominent glomerular lesions in addition to tubulointerstitial injury. The significance of the presence of glomerular lesions in patients with AKI during HFRS is unknown. Therefore, we retrospectively analyzed a cohort of patients with AKI during HFRS with or without glomerular lesions.

## Materials and methods

### Patients

**Figure 1.**
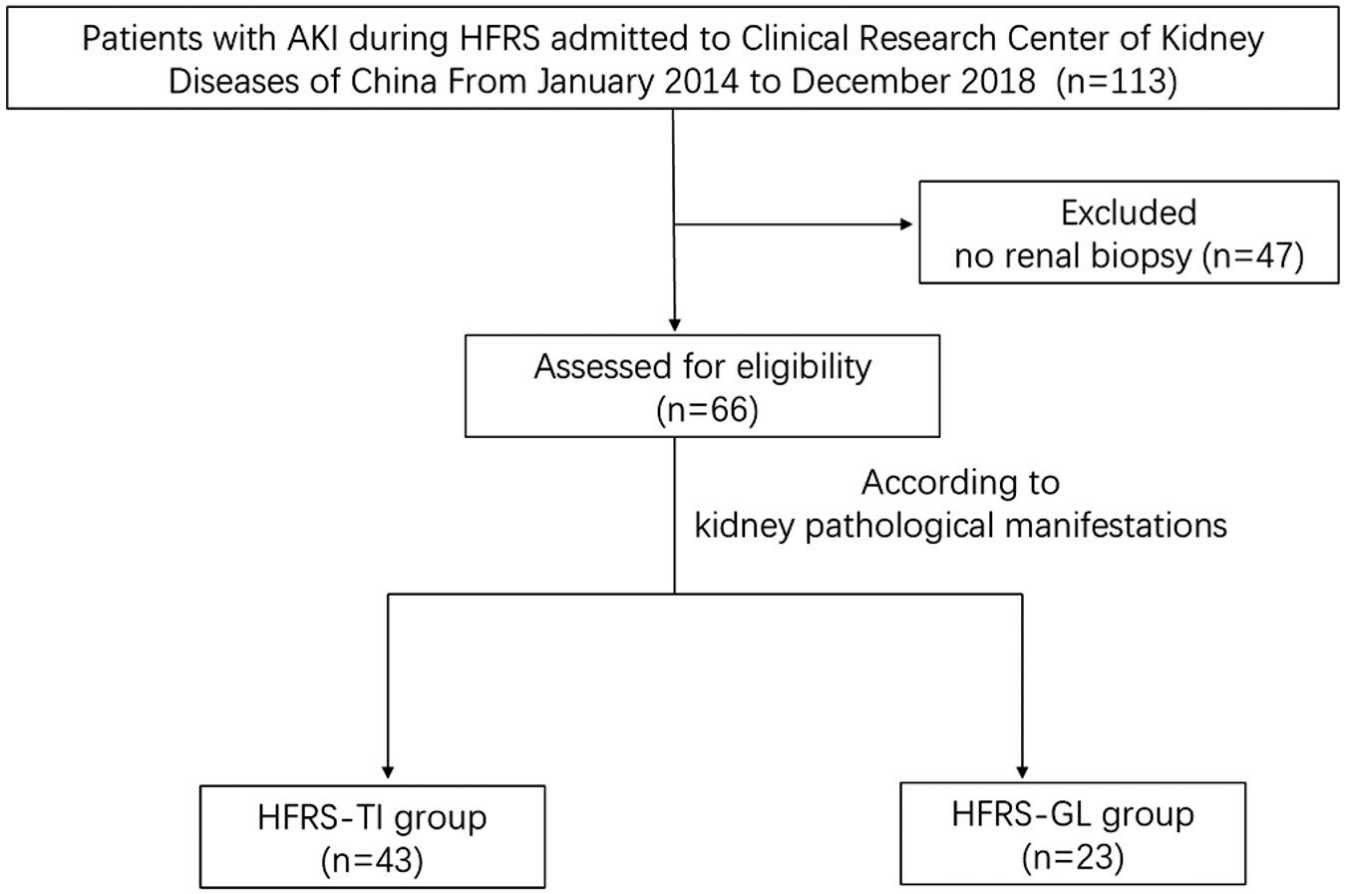
Procedure for screening patients.

We collected 113 patients with AKI during HFRS who sought care at the National Clinical Research Center of Kidney Diseases of China, Jinling Hospital, from January 2014 to December 2018, from which 66 patients who underwent kidney biopsies were selected. According to the pathological findings of the kidney biopsy, the 66 patients were divided into the simple tubulointerstitial injury group (HFRS-TI group) and the tubulointerstitial injury with glomerular lesions group (HFRS-GL group). Eligible patients are required to meet all of the following criteria: (1) definitive diagnosis of HFRS based on clinical symptoms, exposure histories, laboratory tests, and serum-specific antibody tests; (2) no other significant evidence of an alternative diagnosis; (3) underwent kidney biopsy; (4) no previous chronic kidney diseases; and (5) no other possible causes of AKI, such as infection, hypovolemia, or nephrotoxic drugs. The screening process for patients is shown in Figure 1.

### Clinical, laboratory, and pathological data

Clinical data included sex, age, onset course, time of AKI development, and clinical symptoms (fever, hypotension, shock, oliguria, ‘three redness symptoms’, and ‘three pain symptoms’).

Laboratory examination data included proteinuria, microscopic hematuria, white blood cell count (WBC), platelet count (PLT), hemoglobin (Hb), serum albumin (Alb), globulin (Glb), serum creatinine (Scr), urea nitrogen (BUN), alanine aminotransferase (ALT), and aspartate aminotransferase (AST).

Pathological data included light microscopy (hematoxylin and eosin stain, periodic acid stain, periodic acid-silver methenamine stain, and Masson’s trichrome stain), immunofluorescence, and electron microscopy examinations. Tubulointerstitial injury was graded semiquantitatively and defined by the area involved: mild, <25%; moderate, 26%–50%; and severe, >50%.

### Definition

AKI was defined and staged according to the recommendations from Kidney Disease Improving Global Guidelines (KDIGO) [4]. Hypotension was defined as systolic blood pressure <90 mmHg and/or diastolic blood pressure <60 mmHg. Complete remission was defined as a reduction in serum creatinine to normal (Scr < 109.6 µmol/l). Chronic kidney insufficiency was defined as serum creatinine above the normal range (Scr > 109.6 µmol/l) for more than 3 months. End-stage kidney disease (ESKD) was defined as an estimated glomerular filtration rate (eGFR) <15 mL/(min·1.73 m^2^) or kidney replacement >3 months.

### Statistical analysis

Analysis was performed using SPSS 23.0 statistical software. Quantitative variables with a normal distribution are expressed as the mean ± standard deviation (SD), while nonnormally distributed variables are expressed as the median and interquartile range (IQR). Categorical variables are summarized as absolute frequencies and percentages. For continuous variables, independent t tests were applied for normally distributed variables, and the Mann–Whitney Wilcoxon test was applied for nonnormally distributed variables. For categorical variables, the chi-square test or Fisher’s precision probability test was used. Kaplan–Meier analysis was used for survival analysis. Cox regression was used to identify the risk factors for poor outcomes. *p* < .05 was regarded as statistically significant.

## Results

### Baseline demographic and clinical characteristics

Sixty-six patients who fulfilled the inclusion criteria were included in our study after excluding 47 patients without kidney biopsies. Of the 66 patients, 51 (77.3%) were male and 15 (22.7%) were female, with a male-to-female ratio of 3.4:1. There were 43 (65.2%) patients in the HFRS-TI group and 23 (34.8%) patients in the HFRS-GL group. The age at the time of kidney biopsy was 48.3 ± 12.3 years old in the entire cohort, and the median course of the disease was 9.5 (IQR 7–11.3) d. In the HFRS-TI group (30 males and 13 females), the age at the time of biopsy was 47.2 ± 12.4 years old, and the median course of the disease was 10 (IQR 7–12) d. In the HFRS-GL group (21 males and 2 females), the age at the time of biopsy was 49.9 ± 12.3 years, and the median course of the disease was 9 (IQR 7–10) d. The median time of developing AKI in all patients was Day 4 (IQR 3–6.25) of the disease course. The median time of developing AKI was Day 4 (IQR 3–7) and Day 4 (IQR 3–6) in the HFRS-TI group and HFRS-GL group, respectively.

Fever was the first symptom in 62 (93.9%) patients. Oliguria was present in 43 (65.2%) patients during hospitalization. In the course of the disease, 51 (77.3%) patients had proteinuria, 48 (73.7%) patients had microscopic hematuria, 20 (30.3%) patients had ‘three redness symptoms’, 32 (48.5%) patients had ‘three pain symptoms’, and 9 (13.6%) patients developed hypotension. All 66 patients had AKI on admission, including 3 cases of AKI stage 1, 6 cases of AKI stage 2, and 57 cases of AKI stage 3.

There were more males in the HFRS-GL group than in the HFRS-TI group, and the difference between the groups was statistically significant (92.3% vs. 69.8%, *p* = .047). In terms of clinical data and laboratory data, there were no other significant differences between the two groups. More details on the data sources are provided in Table 1.

**Table 1.** Clinical characteristics of the 66 patients with AKI during HFRS.

	Total (*n* = 66)	HFRS-TI group (*n* = 43)	HFRS-GL group (*n* = 23)	*p* value
Age (years)	48.3 ± 12.3	47.4 ± 12.4	49.9 ± 12.3	.475
Sex, male (%)	51 (77.3)	30 (69.8)	21 (91.3)	.047
Course (days)	9.5 (7–11.3)	10 (7–12)	9 (7–10)	.625
Time of AKI (days)	4 (3–6.25)	4 (3–7)	4 (3–6)	.744
Fever (%)	62 (93.9)	41 (95.3)	21 (91.3)	.909
Hypotension (%)	9 (13.6)	7 (16.3)	2 (8.7)	.632
Oliguria (%)	43 (65.2)	28 (65.1)	15 (65.2)	.993
‘Three redness symptoms’ (%)	20 (30.3)	13 (30.2)	7 (30.4)	.986
‘Three pain symptoms’ (%)	32 (48.5)	20 (46.5)	12 (52.2)	.661
Proteinuria (%)	51 (77.3)	33 (76.7)	18 (78.3)	.889
Hematuria (%)	48 (72.7)	33 (76.7)	15 (65.2)	.316
Urine NAG (u/g·cr)	19.6 (10.7–38.3)	19.7 (12.5–38.2)	19.5 (9.3–46.1)	.589
Urine RBP (mg/l)	2.9 (0.5–13.0)	1.7 (0.4–11.9)	3.2 (0.9–14.6)	.870
White blood cells (×10^9^/L)	8.8 (6.9–12.8)	9.0 (6.9–11.9)	8.2 (6.5–14.2)	.581
Red blood cells (×10^9^/L)	4.2 ± 0.8	4.0 ± 0.8	4.1 ± 0.8	.580
Hemoglobin (×10^9^/L)	123.2 ± 24.0	121.0 ± 23.7	127.3 ± 24.4	.304
Platelets (×10^9^/L)	123 (47.8–215.3)	127 (53–230)	103 (42–209)	.231
Serum creatinine (µmol/L)	5.2 (2.7–7.3)	5.3 (3.2–7.3)	4.8 (2.0–7.2)	.619
Urea nitrogen (mg/dL)	32.6 (21.3–53.4)	30.6 (20.7–66.0)	40.3 (21.7–50.6)	.672
Albumin (g/L)	32.0 ± 6.9	32.1 ± 6.8	31.8 ± 7.3	.848
Globulin (g/L)	27.9 (24.1–31.3)	27.9 (23.6–31.2)	27.7 (24.5–33.9)	.701
ALT (U/L)	55.5 (38.0–99.0)	55 (38–101)	58 (35–95)	.866
AST (U/L)	58 (37.3–101.8)	58 (38–94)	63 (32–128)	.814
Renal replacement therapy (%)	28 (42.2)	18 (41.9)	10 (43.5)	1.000
Complete remission (%)	58 (87.9)	41 (95.3)	17 (73.9)	.032

‘Three redness symptoms’: skin flushing in the face, neck, and upper chest; ‘Three pain symptoms’: headache, orbital pain, and low back pain; HFRS: hemorrhagic fever with renal syndrome; AKI: acute kidney injury; ALT: alanine aminotransferase; AST: aspartate aminotransferase.

### Kidney pathology

Two different pathological manifestations were observed: simple tubulointerstitial injury and tubulointerstitial injury with glomerular lesions. The kidney pathological features of the HFRS-TI group were as follows: (1) Light microscopy showed few glomerular lesions and prominent tubulointerstitial damage. Tubular epithelial cells exhibited hyaline degeneration, necrosis, and shedding. In the early stage, edema, hemorrhage, and scattered inflammatory cells were observed in the tubular interstitium. In the later stage, kidney histology showed interstitial fibrosis. (2) Immunofluorescence showed that 23 (53.5%) patients in the HFRS-TI group had immunoglobulin and/or complement deposition. Sixteen (69.6%) of these patients had small amounts of IgM and/or C3 deposited in the mesangial region and/or in the glomerular capillary loops, 5 patients had small amounts of IgA deposited in the mesangial region, and 5 patients had small amounts of C1q deposited in the mesangial region. (3) Electron microscopy revealed extensive, severe endothelial cell damage at the early stage of the disease and proliferative changes at the later stage of the disease. No dense deposits were observed in any of the 23 patients in the HFRS-TI group.

The kidney pathological features of the HFRS-GL group were as follows: (1) Light microscopy showed significant glomerular lesions in addition to the tubulointerstitial injury described above. (2) Immunofluorescence showed that 22 (95.7%) patients in the HFRS-GL group had immunoglobulin and/or complement deposition. (3) In addition to the typical presentation of patients in the HFRS-TI group, 10 (43.5%) patients in the HFRS-GL group had dense deposits under the electron microscope. Combining the results of light microscopy, immunofluorescence, and electron microscopy, the glomerular lesions of 23 patients in the HFRS-GL group were diagnosed as glomerulonephritis. Nine of the 23 patients had IgA nephropathy (IgAN), 1 had membranous nephropathy (MN), 2 had diabetic nephropathy (DN), and 11 had mesangial proliferative glomerulonephritis (MsGN).

Compared with the HFRS-TI group, the HFRS-GL group had a higher proportion of interstitial fibrosis (56.5% vs. 27.9%, *p* = .022) and more deposition of immunoglobulin and/or complement [IgG (34.8% vs. 0, *p* < .001), IgA (52.2% vs. 39.5%, *p* < .001), C3 (69.6% vs. 11.6%, *p* < .001), and C1q (52.2% vs. 11.6%, *p* < .001)]. There were no significant differences in the proportion of global glomerulosclerosis or the degree of acute tubulointerstitial injury between the two groups. Comprehensive pathological characteristics of the patients are provided in Table 2.

**Table 2.** Renal pathological characteristics of 66 patients with AKI during HFRS.

	Total (*n* = 66)	HFRS-TI group (*n* = 43)	HFRS-GL group (*n* = 23)	*p* value
Light microscope (%)				
Proportion of Global sclerosis				
0	27 (40.9)	19 (44.2)	8 (34.8)	.459
0–25%	35 (53.0)	22 (51.2)	13 (56.5)	.678
25–50%	4 (6.1)	2 (4.7)	2 (8.7)	.909
Segmental sclerosis	3 (4.5)	2 (4.7)	1 (4.3)	1.000
Tubulointerstitial acute lesions				
Mild	38 (57.6)	25 (58.1)	13 (56.5)	.899
Moderate	18 (27.3)	12 (27.9)	6 (26.1)	.874
Severe	10 (15.2)	6 (14.0)	4 (17.4)	.991
Interstitial fibrosis	25 (37.9)	12 (27.9)	13 (56.5)	.022
Inflammatory cell infiltration	64 (97.0)	41 (95.3)	23 (100)	.539
Interstitial hemorrhage	27 (40.9)	17 (39.5)	10 (43.5)	.756
Immunofluorescence (%)				
C3+	19 (28.8)	5 (11.6)*	14 (60.9)	<.001
IgA+	21 (31.8)	5 (11.6)*	16 (69.6)	<.001
IgM+	29 (43.9)	17 (39.5)*	12 (52.2)	.324
IgG+	8 (12.1)	0	8 (34.8)	<.001
C1q	17 (25.8)	5 (11.6)*	12 (52.2)	<.001

*Immunofluorescence showed small amounts of immunoglobin and/or complement deposited in the mesangial region and no electron-dense material deposited under electron microscopy. HFRS: hemorrhagic fever with renal syndrome; AKI: acute kidney injury.

### Treatment and follow-up

All patients received symptomatic treatment and supportive care providing hemodynamic and oxygen support. Twenty-seven (40.9%) patients received continuous renal replacement therapy (CRRT), and 1 patient received peritoneal dialysis. All patients eventually achieved dialysis independence except one IgAN case in the HFRS-GL group. The patient developed ESKD and was treated with maintenance dialysis. Initially, this patient received hemodialysis, and his kidney function did not recover and progressed to ESKD. Peritoneal dialysis was eventually chosen for renal replacement therapy.

**Figure 2.**
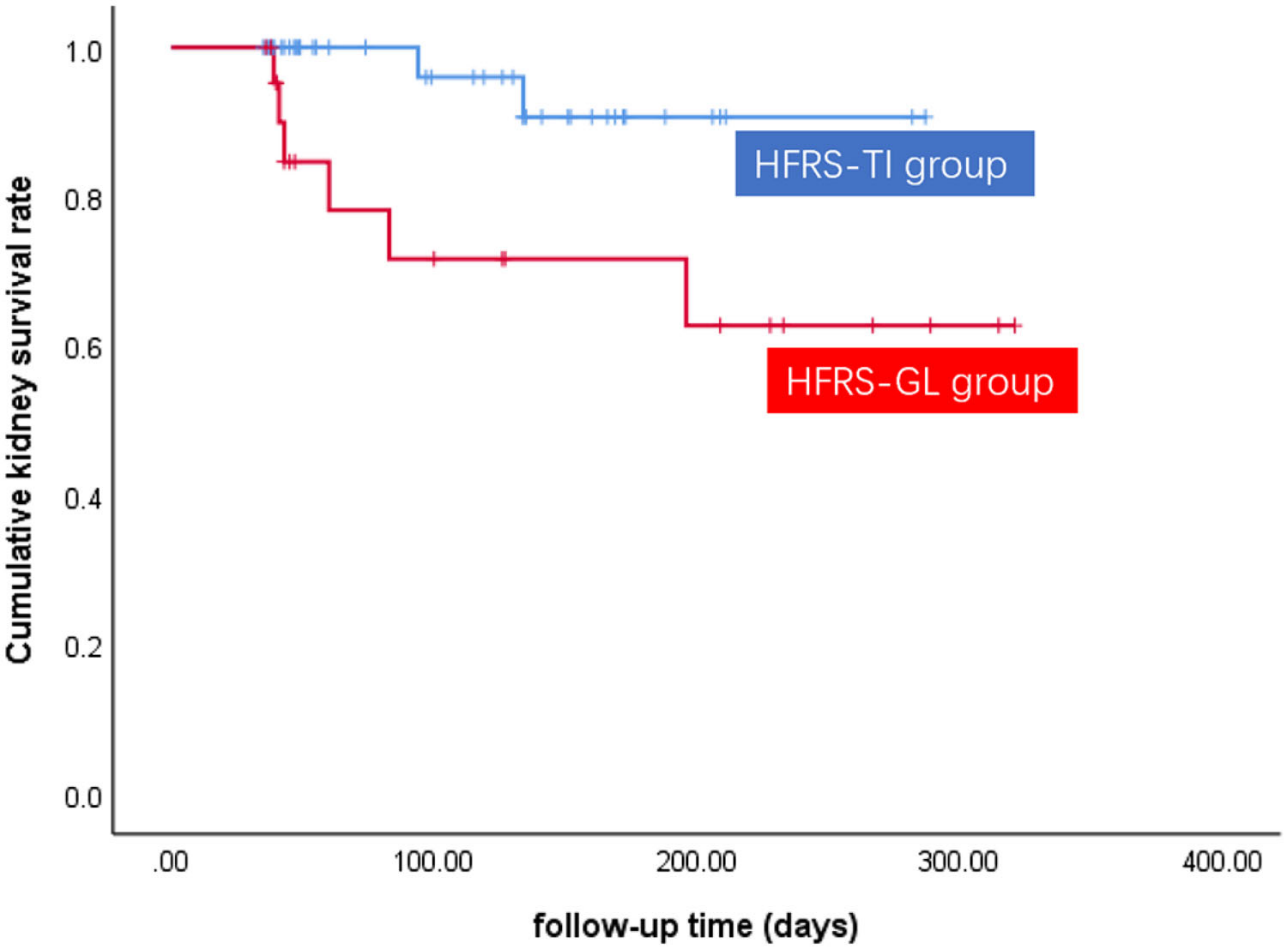
Comparison of the renal survival curve between the HFRS-TI group and HFRS-GL group (log rank test, *p* = .019).

The median follow-up time of the 66 patients was 119 (IQR 43.5–163.5) d. Fifty-eight patients achieved complete remission, and 8 patients eventually progressed to chronic kidney insufficiency (2 in the HFRS-TI group, 6 in the HFRS-GL group). The complete remission rate in the HFRS-TI group was significantly higher than that in the HFRS-GL group (95.3% vs. 73.9%, *p* = .032). Taking chronic kidney insufficiency as an endpoint, we found that cumulative kidney survival in the HFRS-GL group was significantly lower than that in the HFRS-TI group (*p* = .019) (Figure 2). Cox regression analysis showed that moderate tubulointerstitial injury (HR = 3.598, 95% CI = 1.278 ∼ 10.125, *p* = .015) and glomerular lesions (HR = 5.636, 95% CI = 1.121 ∼ 28.329, *p* = .036) were independent risk factors for kidney prognosis.

## Discussion

This is the first study to put forward the view that patients with AKI during HFRS can have glomerulonephritis or prominent glomerular lesions and that patients who have combined glomerular lesions have a worse kidney prognosis than those without. Previous reports suggest that the pathology of kidney damage in HFRS is characterized by acute tubulointerstitial nephritis and can also coexist with glomerular lesions that characteristically show congestion and mild hypercellularity [5]. Interstitial edema and inflammatory cell infiltrations were common clinical manifestations followed by tubulointerstitial alterations. It is worth emphasizing that interstitial hemorrhage is highly suggestive of HFRS. Our findings showed that all of the cases had acute tubulointerstitial injury, 64 of 66 (97%) cases had inflammatory cell infiltration, and edema was found in 38 of 66 (57.6%) cases. Twenty-seven of 66 (40.9%) cases had interstitial hemorrhage. In this study, the low interstitial hemorrhage rate may be due to the sampling error in the medulla, as the hemorrhage is predominantly located in the medulla. Moreover, our study indicates that glomerular lesions play a role that cannot be ignored in the renal damage of HFRS. Mustonen et al. [6] reported that 25% of the kidney biopsy specimens of patients with AKI during HFRS showed slight glomerular mesangial changes, but the relationship between glomerular lesions and kidney prognosis is not clear. Grcevska et al. [7] reported 2 patients with AKI during HFRS who had biopsy-confirmed glomerular lesions. One of the 2 cases was diagnosed as diffuse proliferative glomerulonephritis, and the other was diagnosed as MsGN. The latter progressed to chronic kidney insufficiency. In 2001, Mustonen’s team reported 5 patients with membranoproliferative glomerulonephritis (MPGN) after AKI caused by Puumala hantavirus, and 1 patient developed ESKD [8]. In 2011, they described 7 patients with AKI during HFRS who developed glomerulonephritis proven by biopsy. Of the 7 patients, 5 had MPGN, 1 had MN, and 1 had MsGN. The patient with MPGN developed chronic renal insufficiency during the follow-up period [9]. In our study, 23 of the 66 patients showed obvious glomerular lesions under a light microscope and were diagnosed with glomerulonephritis by a combination of immunofluorescence and electron microscopy. Six of the 23 patients developed chronic kidney insufficiency (1 patient with IgAN developed ESKD). Of the 6 patients, 2 had IgAN, 2 had DN, 1 had MN, and 1 had MsGN. Patients with glomerular lesions had poorer kidney outcomes than those without glomerular lesions. We also showed that moderate tubulointerstitial injury and glomerular lesions in kidney histology are indicators of poor kidney prognosis for patients with AKI during HFRS. These findings suggest that kidney biopsy plays an important role in the diagnosis of kidney involvement in HFRS. Conducting kidney biopsies in patients with AKI during HFRS can help nephrologists predict prognosis, guide subsequent treatment, and help patients establish long-term follow-up for better kidney outcomes.

The relationship between glomerular lesions and HFRS remains unclear. Increased vascular permeability and bleeding caused by vascular endothelial damage are the key pathological changes in HFRS [10]. The insufficiency of effective circulatory blood volume and kidney blood flow leads to a decrease in the glomerular filtration rate. Activation of the renin-angiotensin-aldosterone system, damage to the glomerular basement membrane caused by the virus-induced immune response, and tubular injury are important causes of AKI. AKI during HFRS is an acute process with obvious symptoms. Our study showed that interstitial fibrosis was more prominent in patients with concurrent glomerular lesions, suggesting preexisting glomerular lesions. Two patients in the HFRS-TI group progressed to chronic kidney insufficiency, which indicates the possibility of glomerular lesions arising after HFRS. We hypothesize that hantavirus infection may trigger glomerulonephritis, which may be related to the immune-mediated mechanism of HFRS. A secondary kidney biopsy is required for further confirmation.

In addition, we found that the HFRS-GL group had more male patients than the HFRS-TI group. This may account for female sex being a protective factor against kidney disease [11]. There is a clear male predominance in IgAN and MN [12]. In addition, it has been reported that more male patients suffer from MsGN than female patients. In our study, the cases of IgAN, MN, and MsGN constituted 91.3% of the HFRS-GL group, which was an overwhelming majority. Alternatively, the gender difference between the two groups may be attributed to the small sample size.

The clinical features and severity of HFRS change with age. China has the highest incidence of HFRS. Over the past few decades, approximately 90% of the global cases of HFRS have occurred in China [13]. Typical clinical manifestations of HFRS generally include five clinical stages: fever (3–7 d), hypotension shock (hours to 2 d), oliguria (3–7 d), polyuria (days to weeks), and recovery (2-3 months). The first three stages of severe illness can often overlap; mild cases generally lack the shock or oliguria stage [14]. From the 1960s to the 1980s, the estimate of mortality in HFRS was 5%–15.5% in China, and mucocutaneous injury was the most common manifestation, occurring in more than 90% of patients. Gastrointestinal hemorrhage was the second most common manifestation of HFRS, with an incidence of 50% [15]. According to statistics from the National Health Commission of China and related literature, a total of 1,688,031 cases of HFRS were reported from 1950 to 2020, including 48,260 deaths, with a mortality rate of 2.86% [16]. The mortality rate of HFRS has decreased, as shown by the above statistics. In our study, there were no deaths and no cases of gastrointestinal hemorrhage, and mucosal injury manifested rarely. Most patients present with nonspecific flu-like symptoms, which makes them vulnerable to a misdiagnosis of upper respiratory tract infection or other diseases at the first visit. For patients with kidney injury accompanied by fever, headache, hypotension, thrombocytopenia, liver injury, and other symptoms mentioned in Table 1, HFRS should be considered, and serum etiology confirmation is needed. In the early stage of fever and hypotensive shock, Hantavirus RNA can be detected in the blood of patients with HFRS, and the viral load is positively associated with disease severity [17].

Hantavirus infection is linked to the working and living environment of patients. Humans are not the natural host of Hantavirus, and infection occurs in most cases due to accidental inhalation of virus-containing aerosols in rodent feces. Living or working in an environment with rodents has a high risk of infection [18]. In our study, the patients’ main occupation was farmer or worker, and most of them had experience working outdoors before the onset of HFRS. Additionally, the majority of the patients in this study were male, which is in accordance with the previously reported epidemiological characteristics of HFRS [19]. Rodent control is still the primary measure of prevention of HFRS. Epidemiological surveillance and vaccination are also important measures to protect susceptible populations. Unfortunately, no FDA-approved vaccines or drugs are available. More efforts are needed to develop a usable vaccine or targeted drugs for HFRS. According to previous clinical trials, intravenous ribavirin in the treatment of HFRS significantly reduced the risk of developing severe illness and the risk of mortality, but other studies have not proven these benefits [20].

Early detection, diagnosis, and treatment are the keys to the treatment of HFRS. Fluid therapy and best supportive care are the main treatments in the early stage of the disease. Prevention and treatment of shock, oliguria, bleeding, and other organ injuries are the keys to the successful treatment of HFRS. Patients with kidney failure and severe internal environment disturbance should be treated with timely hemodialysis, and CRRT should be preferred for critical patients with hemodynamic instability.

## Conclusion

The limitation of this study is that it is a retrospective study at a single center. Kidney biopsy was influenced by patients’ subjective intentions. In addition, most of these patients were in critical condition when they presented to our hospital. We acknowledge the study’s limitations, including possible selection bias and information bias.

In summary, patients with AKI during HFRS can have glomerulonephritis or glomerular lesions and are more likely to progress to chronic kidney insufficiency. In addition, moderate renal tubulointerstitial injury indicates poor long-term kidney outcomes. Hence, timely kidney biopsy is of great significance to guide the diagnosis, treatment, and long-term follow-up of patients with AKI during HFRS.
